# Rare case of pure red cell aplasia secondary to smoldering multiple myeloma successfully treated with daratumumab – case report and review of the literature

**DOI:** 10.1007/s00277-025-06218-z

**Published:** 2025-02-19

**Authors:** Malak Alharbi, Sawyer Bawek, Ian Lund, Sean T. Glenn, Steven Green, Hamza Hassan, You-Wen Qian, Jens Hillengass

**Affiliations:** 1 Department of Medicine, Roswell Park Comprehensive Cancer Center, Buffalo, 14263 NY USA; 2 Department of Internal Medicine, King Abdul-Aziz University, Jeddah, Saudi Arabia; 3 Department of Internal Medicine, Jacob School of Medicine and Biomedical Sciences, University at Buffalo, Buffalo, NY USA; 4 Department of Cancer Genetics, Roswell Park Cancer Institute, Buffalo, NY USA; 5 Department of Pathology, Roswell Park Comprehensive Cancer Center, Buffalo, NY USA

**Keywords:** Pure red cell aplasia, Smoldering multiple myeloma, Case report, Daratumumab

## Abstract

Pure red cell aplasia (PRCA) is a rare hematological disorder characterized by erythroid hypoplasia and maturation arrest in the bone marrow. We present a case of acquired PRCA secondary to smoldering multiple myeloma (SMM), initially presenting as severe anemia requiring multiple blood transfusions. This case highlights the diagnostic dilemma at presentation as well as the therapeutic challenges in treating PRCA secondary to SMM. Here we discuss the appropriate workup and identify a potential option for managing these patients with subcutaenous daratumumab.

## Introduction

PRCA is a rare disorder characterized by erythroid hypoplasia and maturation arrest in the bone marrow [1]. PRCA can either be congenital or acquired and may be primary (idiopathic) or secondary to other conditions [2]. Typically, secondary acquired PRCA is associated with collagen, vascular, autoimmune disorders such as systemic lupus erythematous, lymphoproliferative disorders, infections such as parvovirus B19, thymomas, other solid tumors such as gastric, breast, lung, thyroid, and renal cancer, hematologic malignancies, or the use of drugs or toxic agents [1–3]. There have been a few case reports where PRCA has been associated with plasma cell proliferative disorders, in particular, monoclonal gammopathy of undetermined significance (MGUS) and multiple myeloma (MM) [4–10].

Plasma cell proliferative disorders encompass a spectrum of conditions that include MGUS, smoldering multiple myeloma (SMM), and MM. In 1980, SMM was recognized as a distinct entity in the continuum of plasma cell proliferative disorder [11]. According to the International Myeloma Working Group (IMWG) criteria, SMM is distinguished from MGUS by the level of M-protein *≥* 3 g/dL or urinary M protein ≥ 500 mg/24 h and/or the percentage of bone marrow plasma cells (*≥* 10% - <60%). Further criteria are the absence of amyloidosis or myeloma-defining events, including lytic bone lesions or other evidence of end-organ damage [12]. Observation remains the preferred primary approach for managing low-risk SMM [13].

Daratumumab is a monoclonal antibody that targets the cluster of differentiation (CD) 38 protein, a cell surface protein overexpressed on myeloma cells. Daratumumab has demonstrated both single-agent efficacy as well as synergistic effects and improvement in progression free survial (PFS) and in some trials, overall survival when combined with other active anti-myeloma agents, including lenalidomide and dexamethasone, or bortezomib and dexamethasone [14]. As a result, daratumumab received an accelerated U.S. Food and Drug Administration (FDA) approval in 2016 for use in combination with lenalidomide and dexamethasone, or bortezomib and dexamethasone in MM patients who have received at least one prior therapy [15]. Currently, daratumumab is also used for patients with newly diagnosed MM following proven success in the phase 3 MAIA, PERSEUS trials and ALYCONE trial [16–18]. Additionally, the phase 3 AQUILA trial recently demonstrated its clinical benefit for patients with high-risk SMM [19].

Given the rare incidence, no established treatment recommendations are available for PRCA secondary to SMM. Here, we present a described case of PRCA due to IgG-lambda SMM responding to single-agent daratumumab with complete resolution of anemia and review the relevant literature to provide additional context.

### Case presentation

**Fig. 1:**
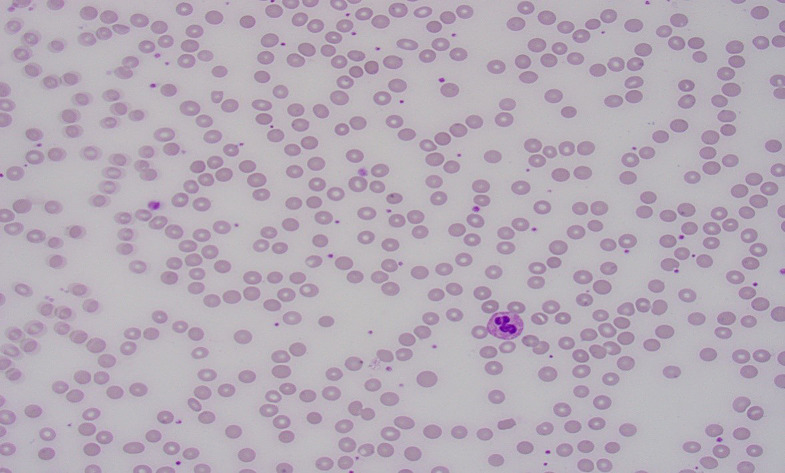
The peripheral blood smear shows normocytic, normochromic red blood cells without Rouleaux formation or schistocytes. The white blood cell count was within normal with occasional plasmacytoid lymphocytes. Platelets were normal in number with some smaller forms present

A 30-year-old female with no prior hematological, autoimmune, or endocrine history presented with exertional dyspnea, fatigue, and multiple near-syncopal episodes of a few weeks’ duration due to severe anemia. Notably, she required approximately fifteen packed red blood cell transfusions and made numerous visits to the emergency department due to symptomatic anemia. She was not on any medications or herbal supplements and had no history of a preceding viral illness. Physical examination findings were notable only for conjunctival pallor. Laboratory workup was remarkable for macrocytic anemia with a hemoglobin of 4.2 g/dL, mean corpuscular volume of 106.8 femtoliters (fl.), reticulocytopenia with a reticulocyte count of 0.8%, absolute reticulocyte counts of 19 × 10^9^/L. Additional results included a white blood cell count of 4.03 × 10^9^/L, platelet counts of 213 × 10^9^/L, and an elevated erythropoietin level of 1504 mIU/ml. She had a significant elevation in inflammatory markers with elevated ferritin of 324 ng/mL, c-reactive protein of 6.97 mg/dL, and erythrocyte sedimentation rate of 69 mm/hr. Initial laboratory workup was negative for hemolytic anemia, autoimmune disease, nutritional and viral etiologies, including parvovirus. The peripheral blood smear showed normocytic normochromic red blood cells without rouleaux formation or schistocytes, normal white blood cells with occasional plasmacytoid lymphocytes, and normal platelets with some smaller forms present (Fig. 1)

**Fig. 2:**
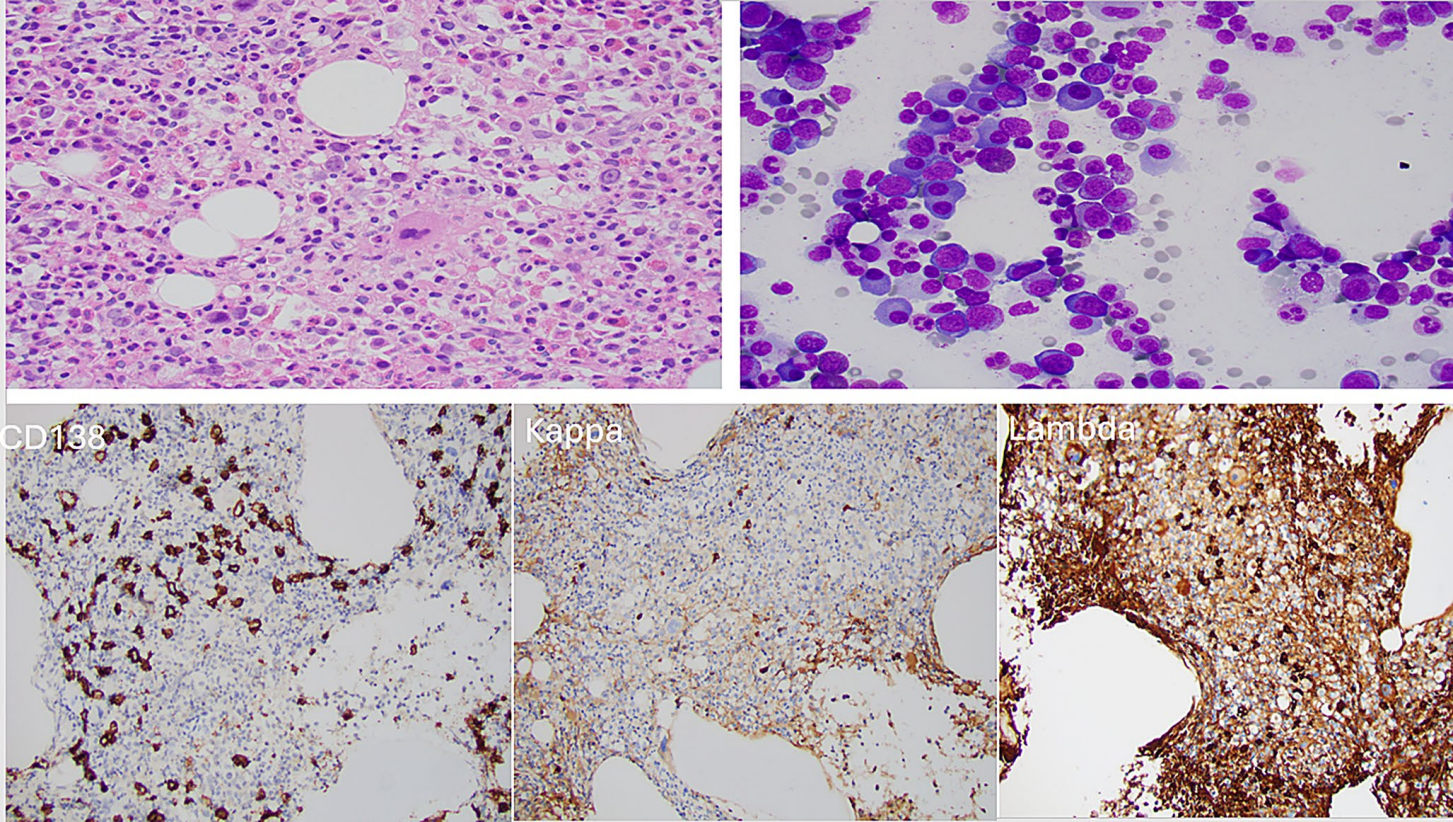
At the top left corner, the bone marrow biopsy showed hypercellular marrow (80% cellularity) with virtually no erythroid islands. The marrow cellularity comprised progressive maturing myeloid cells and an adequate number of megakaryocytes, without lymphoid aggregates. At the top right corner, the bone marrow aspirate smear showed many (7%) atypical plasma cells, some plasma cells had nucleoli and binucleation but overall were mature in appearance. No increased plasma blasts were noted. Myeloid cells were progressively maturing without overt dysgranulopoiesis. Erythroid hypoplasia with rare erythroid precursors. CD138 staining showed about 15% plasma cells that were lambda monoclonal. The plasma cells were negative for CD56, CD117, and B-cell leukemia/lymphoma 1 (BCL1) (not shown)

Notably, serum protein electrophoresis showed a monoclonal protein (M-spike) of 1.87 g/dL; serum immunofixation electrophoresis (IFE) identified IgG lambda. Other results included an IgG levels 2338 g/L, quantitative kappa free light chain (KFLC) of 14.0 mg/L, quantitative lambda free light chain (LFLC) of 774.9 mg/L, and a free light chain ratio of 55.35. An fludeoxyglucose-positron emission tomography (FDG-PET) scan showed no hypermetabolic or individually destructive lesions. Bone marrow biopsy showed a hypercellular marrow (80% cellularity) with virtually no erythroid islands. The marrow cellularity comprised progressive maturing myeloid cells and an adequate number of megakaryocytes with no lymphoid aggregates. The marrow aspirate smears showed 7% atypical plasma cells, some of which were described to have nucleoli and binucleation but overall were mature in appearance with no increased plasma blasts. Myeloid series were progressively maturing without overt dysgranulopoiesis. Erythroid hypoplasia/aplasia with rare erythroid precursors was noted. CD138 staining showed about 15% plasma cells that were lambda monoclonal. By immunohistochemistry, the plasma cells were negative for CD56, CD117, and B-cell leukemia/lymphoma 1 (BCL1) (Fig. 2).

Conventional cytogenetics showed a normal female karyotype in two cells 46 XX [2]. Fluorescence in situ hybridization (FISH) for CD138^+^ enriched plasma cells was unsuccessful. Next-generation sequencing detected a *CREBBP S302N* gene mutation at a variant allele frequency (VAF) of 47.3%. A myeloma minimal residual disease (MRD) panel was performed on an 8-color flow cytometer. A population of monoclonal plasma cells was identified expressing CD38, CD138, CD56, CD81(d), CD27(d), CD45(d), cytoplasmatic lambda light chain, CD86(d), CD200; and was negative for CD19, CD117, and CD28. This population represented approximately 0.9% of total cells, based upon a boolean gating strategy. B cells exhibited a spectrum of differentiation; mature forms were polytypic. The T cell CD4:CD8 ratio was 1.2. Sparse myeloblasts were measured at approximately 0.8% of total cells based on the CD34^+^ percentage.

### Treatment

SMM-associated PRCA was the leading proposed diagnosis, and a decision was made to start the patient on the CD38-targeting agent daratumumab-hyaluronidase 1800 mg subcutaneous and dexamethasone 40 mg according to clinical standards. The patient additionally received methylprednisolone post-daratumumab after the first cycle to decrease the risk of infusion reactions. Follow up laboratory results after the first cycle showed a partial response, with a reduction in serum monoclonal protein from 2.29 g/dL to 0.95 g/dL. Additionally, hemolgobin levels improved from 6.3 to 8.1 g/dL. The patient had so far completed 12 cycles of daratumumab/dexamethasone with a dose reduction of the latter to 20 mg once a month due to side effects. She had achieved a complete count recovery with resolution of the anemia and most recent hemoglobin of 15.0 g/dL. Furthermore, she achieved deep remission, defined as very good partial response (VGPR) with IFE positivity, negative M-spike, and 1% bone marrow plasma cells without signs of clonality. MRD was negative by flowcytometry at 10^− 5^. Given achievement of VGPR, and negative MRD, it was decided to stop the treatment after a duration of one year. Table 1 shows clinical response.

## Discussion

PRCA is characterized by selective erythroid hypoplasia and maturation arrest [6]. Secondary PRCA associated with plasma cell neoplasms, such as SMM in this case, presents a unique challenge due to its rarity, complicating the understanding of anemia and maturation arrest mechanisms. In our patient only 15% plasma cell involvement was observed in the bone marrow, which was disproportionate to the severity of her anemia. The diagnosis of secondary PRCA was further supported by a low reticulocyte index and the scarcity of erythroid progenitors in the bone marrow. Additioanlly, other potential etiologies of anemia, incluidng hemolysis, nutritional deficiencies, infections, and bleeding disorders, were excluded. This led to the decision to manage the patients as having SMM with PRCA rather than MM with a myeloma-defining event of anemia.

While plasma cell proliferative disorders in young patients are uncommon, they have been documented, mainly in the context of MM. SMM in young patients is exceedingly rare [20, 21]. Initially, a myelodysplastic neoplasm was considered in the differential diagnosis due to its association with PRCA [2, 22, 23]. However, no characteristic clonal abnormalities or other suggestive features, such as dysplasia, were observed in the patient’s case. While *CREBBP/EP300* gene mutations have been described in association with myeloid neoplasms [24]. However, the ClinVar interpretation of the *CREBBP S302N* variant is benign or likely benign, and the ~ 50% VAF suggests it may be a constitutional variant of uncertain significance rather than indicative of a clonal process [25]. Given the severity of the anemia and the inability to attribute it to causes other than the SMM, treatment initiation was deemed necessary, despite not meeting the criteria for high-risk SMM.

A retrospective study analyzing 1363 patients with newly diagnosed MM showed that 84% of patients had anemia at the time of diagnosis. Among these, 753 out of 1,363 had moderate anemia (hemoglobin “hgb” 9.0–12.0 g/dL) or severe anemia (hgb 6.0–9.0 g/dL) [26]. The multiple mechanisms causing anemia in MM and the replacement of the physiological bone marrow by malignant cells, are not yet fully understood. One contributing factor in some patients is reduced erythropoietin level, due to renal insufficiency resulting from MM [27]. Furthermore, changes in the tumor microenvironment have been described as a contributing to the development of anemia [28]. Another proposed theory is impairment of iron utilization due to the hepcidin upregulation in patients with MM, which occurs as a result of elevated interleukin-6 (IL-6) levels [29, 30]. In another retrospective analysis of 25 patients with MM, serum hepcidin levels were singnificanlty higher compared to age-matched healthy population (*P* < 0.01). Among patients with MM with normal kidney function (*n* = 16), serum hepcidin levels were found to correlate inversely with hemoglobin concnetratation (*P* = 0.006), suggesting a link between hepciding dysregulation and the severity of anemia [31]. Furthermore, IL-6 production correlated with disease severity due to the autocrine production from the immature CD45^+^ MM cells [32, 33]. The patients in the study had 15% lambda-predominant plasma cells.

Additionally, a study showed co-culturing normal donor hematopoietic stem cells (HSCs) with serum from a PRCA patient containing M-protein led to a 43% reduction in colony-forming unit-erythroid lineage (CFU-E) and burst-forming unit-erythroid (BFU-E) compared to serum from a normal donor, suggesting inhibition of erythroid colonies by the monoclonal protein. Presumably, this is due to antibody recognition of an antigen on erythroid precursors. Interestingly, it was also reported that one patient experienced a resolution of anemia after being treated with bortezomib-based therapy [6].

Considering the patient’s young age and desire to preserve fertility, we engaged in a discussion regarding the initiation of treatment with daratumumab versus lenalidomide, ultimately choosing daratumumab. At the time of patient presentation, there was no established guidelines for duration of use of daratumumab in patients with SMM. Recently Dimopolous et al. published phase 3 AQUILA study which investigated daratumumab administration for up to 36 months, versus survilalnce in patients with high-risk SMM. This study had a fairly borad definition of high risk disease which would have included our study patient given free light chain ratio over 8. The daratumumab arm showed a significant improvement in PFS (estimated 60-month PFS 63.1% versus 40.8%) in surveillance group, as well as time to first-line SMM treatment [19]. As there was no published precedent for the use of daratumumab in SMM at that time, we decided to stop daratumumab after 12 cycles given excellent disease response and complete resolution of anemia.

We summarized published case reports of patients with monoclonal plasma cell disorders and PRCA in (Table 2). A recently published case by Michel CS and colleague, described a similar young patient presenting with severe anemia due to monoclonal gammopathy-PRCA refractory to multiple lines of therapy [10]. This patient initially responded to anti-CD38 daratumumab; however, the patient experienced disease progression while on maintenance daratumumab, manifesting as worsening PRCA and an elevation of the M-spike. Notably, this patient eventually responded to another CD38 targeting agent, isatuximab [10] (Table 2). Unlike the aforementioned case, our patient demonstrated an immediate response to daratumumab, and had a higher percentages of plasma cells at time of diagnosis. These cases underscore the importance of recognizing the diverse manifestations of plasma cell dyscrasias and highlights the necessity of personalized medicine in tailoring treatment strategies to individual patients.

Anti-CD38 monoclonal antibodies, including daratumumab and isatuximab, have been successful in treating refractory PRCA following allogeneic hematopoietic stem cell transplantation (aHSCT) with major ABO incompatibility [34–38]. This further support the notions that targeting CD38^+^ cells directly may facilities red blood cell recovery and help control PRCA by reducing red-cell antibody production. These observations call for further research into the underlying mechanisms of anemia in plasma cell dyscrasias, as well as the changes occurring in the microenvironment with CD38-targeting agents, to better inform clinical management and improve patient outcomes.

**Table 1:** Trends in hemoglobin levels, serum M-spike, free light chain ratio, and bone marrow plasma cell percentage during treatment

Laboratory	Prior to C1^+^	End of C1	End of C3	End of C5	End of C7	End of C9	End of C12
Hemoglobin g/dL	4.2	10.1	14.8	14.2	14.7	13.9	15
Serum IgG lambda, M-spike: g/dL	2.56	0.95	0.52	0.35	0	0	0
Free light chain ratio (0.26–1.65)	45.5	6.12	1.94	1.96	1.56	1.37	1.27
% of plasma cell in the bone marrow	15%						1%, MRD negative

^+^ Treatment with daratumumab 1800 mg subcutaneous and dexamethasone 40 mg

M-spike; monoclonal protien, C1; treatment cycle 1, C3; treatment cycle 3, C5; treatment cycle 5, C7; treatment cycle 7, C9; treatment cycle 9, C12; treatment cycle 12, MRD: minimal residual disease by flowcytometry

**Table 2:** Review of the literature: monoclonal plasma cell disorders associated with PRCA: clinical presentation, treatment, and outcomes

Patient	Age (years)	Subtype	SerumM-spike (g/dL)	Free light chain ratio	BM examination	Hgb g/dL	Reticulocyte counts	Treatment	Response	Ref
1	44	IgG lambda IgA lambda	NA	2.85	14% PC and lack of erythropoiesis.	3.4	0.05%	PAD (anemia persisted) thus switched to cyclosporine	resolution of anemia	[4]
2	41	IgG lambda	9.8	NA	12% PC, absent erythroblasts	NA	NA	cyclosporine rituximab	resolution of anemia on rituximab, VGPR	[5]
3	48	IgG kappa	NA	11.87	20% PC, 8% erythroid precursors	6.4	6.9	VD	resolution of anemia and decrease in M spike	[6] (patient #2)
4	57	IgG lambda	NA	0.92	20% PC, 4% erythroid precursors	8.6	15.4	RVD	resolution of anemia	[6] (patie# 8)
5	67	IgG lambda	NA	0.45	9% PC, 3% erythroid precursors	8.0	6.4	LD	resolution of anemia and CR	[6] (patient #12)
6	29	IgG	NA	NA	20% PC, < 2% erythroid precursors	7.6	NA	VAD cyclosporine methylprednisolone cyclophosphamide + ATG plasma exchange	PD	[7]
7	61	IgG	0.44	NA	6% PC, erythroid hypoplasia	3.7	0.01	IVIG followed by cyclosporine and prednisone. VD	resolution of anemia and disappearance of M spike with VD	[8]
8	55	IgG lambda	5.1	NA	7% PC, absent erythroid precursors	NA	NA	NA	NA	[9]
9	44	IgG Lambda	6	2.3	< 5% PC, absent erythroid precursors	9.0	0.09%	VD, LD cyclosporine, alemtuzumab dara-VD, KD, ID, Isa-P	VGPR	[10]
10 Indexed Patient	30	IgG lambda	2.56	45.4	15% PC, absent erythroid precursors	4.2	19 × 10^9^/L	daratumumab and dexamethasone	resolution of anemia, VGPR	N/A

++ After 3 cycles of daratumumab, bortezomib and dexamethasone (Dara-Vd), the patient achieved very good partial response (VGPR) with a reduction in M-spike reduction to 1.5 g/L. However, both M-spike and anemia worsened during maintenance daratumumab prompting a switch to carfilzomib and dexamethasone (KD) without response. The patient then responded to isatuximab, pomalidomide, and dexamethasone (Isa-P), showing a decrease in M-spike and resolution of anemia

M-spike: monoclonal protein; BM: bone marrow; Hgb: hemoglobin; Ref: reference; NA: not available; PC: plasma cells; PAD: bortezomib, doxorubicin and dexamethasone; VGPR: very good partial response; VD: bortezomib and dexamethasone; RVD: lenalidomide, bortezomib and dexamethasone; LD: lenalidomide and dexamethasone; CR: complete remission; VAD; vincristine, doxorubicin and dexamethasone; ATG: anti-thymocyte globulin; PD: disease progression; IVIG: intravenous immunolglobulins; Dara-VD: daratumumab, bortezomib and dexamethasone; KD: carfilzomib and dexamethasone; ID: ixazomib with dexamethasone; Isa-P: isatuximab, pomalidomide, and dexamethasone, N/A: not applicable.

## Data Availability

No datasets were generated or analysed during the current study.
